# The relationship between cross-reactive Plasmodium vivax antibody responses and P. knowlesi parasitaemia and clearance

**DOI:** 10.21203/rs.3.rs-11044112/v1

**Published:** 2026-10-11

**Authors:** Jeyamalar T. Thurai Rathnam, Megha Rajasekhar, Rhea J. Longley, Anju Abraham, Timothy William, Sitti Saimah Sakam, Daniel J. Cooper, Giri S. Rajahram, Bridget E. Barber, Nicholas M. Anstey, Matthew J. Grigg, Julie A. Simpson

**Affiliations:** The University of Melbourne; The University of Melbourne; Walter and Eliza Hall Institute of Medical Research; Walter and Eliza Hall Institute of Medical Research; Infectious Diseases Society Sabah-Menzies School of Health Research Clinical Research Unit; Infectious Diseases Society Sabah-Menzies School of Health Research Clinical Research Unit; Menzies School of Health Research; Infectious Diseases Society Sabah-Menzies School of Health Research Clinical Research Unit; Menzies School of Health Research; Menzies School of Health Research; Menzies School of Health Research; The University of Melbourne

**Keywords:** Malaria, Plasmodium knowlesi, Plasmodium vivax, parasite count, antibodies, cross-species immunity, parasite clearance

## Abstract

**Background:**

Pre-existing antibodies to *Plasmodium vivax* may cross-react with *P. knowlesi* and influence infection outcomes. We investigated whether baseline *P. vivax* antibody levels affect parasitaemia and clearance in knowlesi malaria.

**Methods:**

Total IgG to 21 *P. vivax* antigens was quantified in pre-treatment plasma from patients in three Malaysian clinical trials. Parasitaemia was assessed 6-hourly by microscopy and clearance half-life estimated. Antibody associations with *P. knowlesi* baseline parasitaemia and clearance were identified using regression and machine learning approaches (elastic net regression and random forests).

**Results:**

Data were available for 134 patients (median age 37 years, 37% with prior malaria). Five antibodies were associated with reduced baseline *P. knowlesi* parasitaemia (0.75–0.90-fold lower per two-fold increase, 95% CIs ranging from 0.65–1.00). Median *P. knowlesi* parasite clearance half-life was 2.6 hours (IQR 2.1–3.2). Two antibodies were associated with slightly slower parasite clearance (1.04-fold increases in mean clearance half-life per two-fold antibody level). Within-group correlation analyses revealed widespread, positive antibody associations in *P. knowlesi* infections, with weaker but coordinated (RBP and DBP family) responses in malaria-negative endemic controls.

**Conclusions:**

Cross-reactive *P. vivax* antibodies may partially constrain *P. knowlesi* parasitaemia, but functional studies are needed to clarify their role in immunity and zoonotic transmission.

## Background

The monkey parasite, *Plasmodium knowlesi*, causes zoonotic malaria in humans across Southeast Asia, and can result in severe disease and death [1–3]. Over the last 15 years, Malaysia has seen the decline and elimination of indigenous *P. falciparum* and *P. vivax* transmission [4–7]. However, over the same period, there has been an increased incidence of zoonotic *P. knowlesi* infections [4, 8], with over 22,000 reported cases and 74 deaths from 2017–2024 in Malaysia [7, 9]. This relative change in species predominance, and the close genetic relationship between *P. vivax* and *P. knowlesi* parasites [10], raises the possibility that the decrease in *P. vivax* transmission and potential loss of cross-protective host immunity may have contributed to the rising susceptibility to *P. knowlesi* infection and/or disease [11]. Immunoglobulins generated by the host adaptive immune response against *P. falciparum* and *P. vivax* are robust indicators of the degree of previous exposure [12, 13]. They have been shown to confer protection against both uncomplicated and severe clinical disease, as well as high blood-stage parasite densities, however only in subsequent infections with the same species [14–20]. In contrast, some evidence does suggest cross-species protection between *P. vivax* and *P. knowlesi*, with their close genetic relationship including homology in proteins involved in red blood cell invasion by merozoites in both species [21]. Naturally acquired human IgG antibodies to *P. vivax* proteins orthologous to *P. knowlesi*, such as Pv41 and Pv-Rhop-H2 (representing merozoite surface and apical organelles respectively), inhibit *P. knowlesi* parasite growth *in vitro* in a concentration-dependent manner [22]. Similarly, historical malariotherapy studies indicate that patients previously infected with *P. vivax* were more resistant to developing symptomatic *P. knowlesi* infections after direct inoculation [23]. It has also been shown that higher *P. falciparum*-specific antibody levels coupled with phagocytosis of merozoites are associated with faster parasite clearance for falciparum malaria [24], however, it is unclear if the same relationship exists for *P. vivax* or *P. knowlesi* infections.

Understanding the cross-reactivity of immune responses to *P. vivax* could suggest strategies for deployment of future *P. vivax* vaccines to provide concurrent protection against zoonotic malaria [2], including severe infections with high parasitaemia. This study used a diagnostic panel of *P. vivax* antigens to delineate and measure the potential effect of pre-existing anti-*P. vivax* antibodies on *P. knowlesi* parasitaemia and parasite clearance.

## Methodology

### Patients

Previously generated *P. vivax* IgG antibody data from patients with *P. knowlesi* malaria were available from a published diagnostic serological cross-reactivity study [25]. This study included samples collected on day 0 prior to treatment from three *P. knowlesi* clinical trials (ACT KNOW [26], CAN KNOW [27], and PACKNOW [28, 29]) conducted in Sabah, Malaysia between 2012 and 2018, a period spanning the elimination of local autochthonous transmission of the human-only species *P. falciparum* and *P. vivax* in Sabah [6]. The ACT KNOW and CAN KNOW trials included children and adults with uncomplicated knowlesi malaria, similar to the PACKNOW trial which additionally included severe *P. knowlesi* malaria (Supplementary Table 1). PCR [30, 31] confirmed *P. knowlesi* mono-infection in all patients, and excluded *P. vivax* co-infection at enrolment. Data on self-reported prior episodes of malaria were available; however, the *Plasmodium* species was unknown. Across all clinical trials, parasitaemia was measured using microscopy at enrolment (time 0) and then six-hourly following antimalarial treatment until the parasites were no longer detectable.

### Determination of antibody responses to P. vivax antigens

Briefly, 21 *P. vivax* proteins (Supplementary Table 2) were previously chosen for diagnostic utility based on the individual ability of antibodies to these proteins to classify recent or chronic subpatent *P. vivax* exposure [13] or because they provided complementary information on another protein (e.g. MSP3b for MSP3a). Total IgG levels against these 21 *P. vivax* antigens were measured by Luminex xMAP beadbased immunoassay, and expressed as relative antibody units (RAU) based on normalisation against a 5point serial dilution curve, as previously described [25]. Of the 21 *P. vivax* antibodies chosen, three (antibodies to the *P. vivax* rhoptry protein RAMA, and the surface proteins, MSP8 and MSP1–19) have been previously shown to cross-react with *P. knowlesi* proteins and to be associated with 30–50% inhibition of *P. knowlesi* RBC invasion *in vitro* [22].

### Healthy control groups from endemic and non-endemic malaria areas

Antibody data for malaria naïve controls were provided by 369 blood donors from non-malaria endemic urban areas with no reported previous malaria history from Bangkok (Thailand), Rio De Janeiro (Brazil) and Melbourne (Australia) [13, 25]. To provide in this study an additional malaria-endemic matched control group reflecting similar prior population-level exposure risk, antibody responses were assessed in 112 non-febrile, *Plasmodium* PCR-negative controls from Sabah, Malaysia, with unknown malaria history.

### Statistical analysis

The *P. knowlesi* parasite clearance half-life was estimated for each patient using the WorldWide Antimalarial Resistance Network Parasite Clearance Estimator (WWARN PCE) tool [32–34]. The association between day 0 total IgG levels against 21 *P. vivax* antigens and *P. knowlesi* baseline parasitaemia (pre-treatment) was evaluated using linear regression analyses for each antigen, adjusting for age and sex. The association between day 0 total IgG levels against 21 *P. vivax* antigens and parasite clearance half-life was evaluated using separate linear regression analyses for each antigen, in four ways, with and without age and sex adjustments, and by type of antimalarial treatment (artemisinincombination therapy or chloroquine), respectively. Antibody levels were log_*2*_ transformed and baseline parasitaemia and clearance half-lives log_*e*_ transformed due to skewness. To further identify antibodies associated with *P. knowlesi* baseline parasitaemia (pre-treatment) and parasite clearance half-life, two machine learning approaches were applied, elastic net regression and random forests [35–38], with antibody features standardized (mean 0, SD 1) and collinearity assessed using variance inflation factors (threshold = 5). Leave-one-out cross-validation (LOOCV) was used for internal validation, with model performance assessed by mean squared error (MSE). Variable importance was derived from regression coefficients (elastic net), and percentage increase in MSE (random forests). Analyses were conducted in R v4.1.2 [39] using the *caret* [36–38], *glmnet* [40, 41], and related packages, with the aim of identifying antibody features most predictive of baseline parasitaemia and parasite clearance.

Further descriptive analyses were performed to distinguish acute infection-driven from pre-existing antibody responses. Pairwise spearman correlations of log_2_ transformed antibody levels for each antigen were calculated separately for *P. knowlesi* (day 0) cases, endemic malaria-negative controls from Sabah and non-endemic malaria naïve controls.

## Results

A total of 134 out of 140 patients with previously generated serological data were included in this analysis; 3 patients were excluded because there were no baseline (day 0) antibody measurements available and 3 patients were excluded because parasite clearance half-life could not be estimated. The available data were from 100 males and 34 females with a median age of 37 (range 3–78) years (Table 1). Of these patients, 37% reported having had a previous malaria episode. Patients were treated with oral chloroquine (34%) or ACT with or without additional intravenous artesunate (66%). The median (inter-quartile range) parasite count at presentation was 2,773/μL (662–8,163) and parasite clearance half-life was 2.6 hours (2.1–3.2).

Across all 134 patients with knowlesi malaria, pre-treatment total IgG levels to the 21 tested antigens ranged from 0.00001 to 0.02 relative antibody units (RAU), as previously described [25]. Among these, the strongest response was to merozoite surface protein (MSP1.19), while the weakest was to erythrocyte binding protein (EBP) (Supplementary Fig. 1). For comparison, baseline antibody responses for 112 healthy controls from malaria-endemic areas in Sabah showed higher median RAU compared to the malaria-naïve controls for most of the antigens, except EBP and MSP8. DBPII_Sal1 responses were lower in *P. knowlesi* malaria patients compared to healthy Sabah controls, although higher than those in malaria-naïve individuals (Supplementary Fig. 1).

The median antibody levels at baseline for the two major treatment groups used in this study, artemisinin-based and chloroquine, were comparable (Supplementary Table 3). The median parasite count at baseline for each of the treatment groups were similar, with a median of 2774/ μL (IQR: 748–10154) and 2606/ μL (IQR: 662–6356) for patients treated with ACT and chloroquine respectively. The parasite clearance half-lives, however, differed across the two groups, with ACT (with or without intravenous artesunate) having a median of 2.4 hours (IQR: 2.0–3.0) compared to 3.1 hours (IQR: 2.6–3.8) in the chloroquine group.

Antibodies to five *P. vivax* antigens measured on day 0 were associated with a lower mean *P. knowlesi* parasite count at baseline, after adjustment for age and sex (Table 2). Two-fold higher antibody levels to these five *P. vivax* antigens were associated with a lower estimated geometric mean baseline *P. knowlesi* parasite counts ranging from 0.75-fold (95% CI: 0.65, 0.86) to 0.90-fold (95% CI: 0.82, 1.00) (Table 2 and Fig. 1A). These antibodies included MSP-8 and RAMA which have been previously associated with a 50% (MSP-8) and 40% (RAMA) inhibition of *P. knowlesi* red blood cell invasion *in vitro* [22].

For the outcome *P. knowlesi* parasite clearance half-life, antibody responses to two *P. vivax* antigens (MSP7L and DBPII_AH) were associated with a small increase of 1.04-fold in the geometric mean parasite clearance half-life (Supplementary Table 4 and Fig. 1B). Due to the differing effect of ACTs and chloroquine on parasite clearance in these patients, analyses were stratified by treatment group. Results were generally similar between groups, although antibodies to one antigen (MSP3b) was associated with slower parasite clearance in patients treated with chloroquine (Supplementary Table 5).

Antibody measurements for all 21 *P. vivax* antigens at baseline (day 0) were analysed simultaneously using each of the two machine learning methods outlined above. The inclusion of antimalarial drug resulted in improved model performance in all analyses of parasite clearance, consistent with previous findings that ACT clears parasites faster than chloroquine [26, 27, 34].

Elastic net regression using 21 *P. vivax* antibody features and baseline *P. knowlesi* parasitaemia as the outcome identified three antibody features with non-zero coefficients, PVX_096995, PVX_112670 and TRAP (Table 3). These represented a subset of the five antibody features associated with lower baseline parasitaemia in the above univariate linear regression analyses (Table 2). For the outcome parasite clearance half-life, the two antibody features selected with non-zero coefficients were MSP7L and DBPII_AH (Table 3), indicating that higher levels of these antibodies were associated with longer *P. knowlesi* parasite clearance half-lives. The resulting model performance, indicated by the square root of the mean squared error (RMSE), was 0.499. The same 21 antibody features were then analysed together with demographic factors potentially associated with parasite clearance (age, sex, treatment and baseline parasitaemia). This led to slightly improved model performance with a RMSE of 0.483 (Table 3) and a subset of five chosen antibody features.

The random forest method was used on all 21 *P. vivax* antibody features, with *P. knowlesi* baseline parasitaemia as the outcome (Table 3), and 8 antibody features were selected as important, with the top 3 being PVX_096995, PVX_112670 and TRAP, also selected by elastic net regression. The same method was performed for the outcome parasite clearance half-life, and as for elastic net regression, the top antibody feature selected was MSP7L.

To further understand the effect of antibody responses on initial parasitaemia and parasite clearance (presented above), descriptive analyses were performed comparing antibody profiles in patients with *P. knowlesi* malaria, malaria-negative endemic controls from Sabah, and non-endemic malaria-naïve controls. Correlation analyses revealed marked differences in antibody co-acquisition across these groups (Fig. 2), with progression from fragmented responses in malaria-naïve individuals to more coherent correlations in endemic controls, particularly among invasion-related proteins including RBP, DBP and merozoite surface proteins (representing pre-existing antibody responses). In acute *P. knowlesi* infections, correlations were broadly stronger across most antibody responses, consistent with widespread boosting of antibody responses during infection.

## Discussion

Cross-reactive antibody responses to almost all of the 21 *P. vivax* antigens assessed in our panel were present in patients presenting with knowlesi malaria, to differing levels dependent on antigen and consistent with previous evidence of shared antigenicity between the two species [25]. In this cohort, higher antibody responses to five of these *P. vivax* antigens were associated with substantially lower pre-treatment *P. knowlesi* parasite counts. Of these, *P. vivax* MSP8 and RAMA have been previously associated with a 50% and 40% inhibition, respectively, of *P. knowlesi* red blood cell invasion *in vitro* [22], while others have not been tested for functional inhibition. These *in vivo* associations provide clinical support for a protective role of cross-species antibodies in constraining parasite growth at the time of acute presentation. In contrast, antibody responses were not associated with faster parasite clearance following treatment. Taken together, these findings suggest that pre-existing cross-reactive *P. vivax* responses may help limit the initial parasite burden in acute *P. knowlesi* infections but are unlikely to influence the rapid, drug-mediated clearance phase of infection.

The five antigens which showed the strongest association with lower baseline parasitaemia were PVX-112670, TRAP, RAMA, PVX_096995, MSP8. The diverse functions of these proteins, including parasite RBC attachment, invasion and intracellular survival may contribute to controlling parasitaemia in acute *P. knowlesi* infection independent of their surrogate exposure status. RAMA and MSP8 have been previously shown by Muh et al. to be cross-reactive with *P. knowlesi* and associated with inhibition of *P. knowlesi* invasion *in vitro* [22]. *P. knowlesi* parasitaemia at the time of clinical presentation is a strong predictor of severe malaria and end-organ dysfunction [42, 27, 43], so the ability of pre-existing antibodies to *P. vivax* to limit parasitaemia has important clinical and epidemiological implications. Lower parasitaemia may reduce the risk of symptomatic or severe *P. knowlesi* malaria[23]. Conversely, declining *P. vivax* incidence in co-endemic areas, including its recent elimination in Malaysia [6], could erode this protective effect and potentially lead to more frequent symptomatic and severe *P. knowlesi* infections [2].

Higher antibody levels showed only weak associations with longer parasite clearance half-lives for two *P. vivax* proteins, MSP7L and DBPII_AH, adjusted for age and sex. However, the magnitude of these associations was small, with a two-fold increase in *P. vivax* MSP7L antibody levels corresponding to only a 1.04-fold longer *P. knowlesi* clearance half-life, equivalent to an increase from 2.6 to 2.7 hours. The biological significance of these findings is uncertain. As the original selection of lower shared sequence identity proteins aimed to improve the specificity of *P. vivax* exposure classification against *P. knowlesi*, it is possible that the most relevant markers of heterologous protective immunity to *P. knowlesi* at baseline were not included. Multivariable model performance improved modestly with the inclusion of clinical covariables, with treatment as the expected strongest predictor of clearance and no clear contribution of host immunity. Unlike *P. falciparum*, where naturally acquired immunity can accelerate clearance even in the presence of kelch13 drug resistance [44], there is currently no *in vitro* evidence of antimalarial resistance in *P. knowlesi* [45], and treatment efficacy remains high in endemic areas with a macaque reservoir [26, 46, 27]. The short median clearance half-life in this cohort (2.6 hours) compared to typical values in *P. falciparum* further constrains the ability to detect antibody-mediated effects. Alternative study designs, such as reinfection cohorts or prospective surveillance of clinical recurrences, may be required to uncover more subtle contributions of cross-protective immunity to parasite clearance dynamics, including in a hypothesized hidden burden of spontaneously resolving infections in higher endemic areas [47].

There is evidence that older patients infected with *P. knowlesi* have an increased risk of severe disease [42, 48], with demonstrated higher proportional innate inflammatory responses and endothelial activation in response to parasite loads [43]; however, older infected patients also have higher seropositivity to immunity biomarkers [49]. A population-based study of households in the northern part of Sabah, Malaysia, found that increasing age was associated with increased seroprevalence for *P. falciparum*, *P. vivax* and *P. knowlesi* [50]. Individuals with high antibody levels were detected in all age groups for *P. knowlesi* and similar to *P. falciparum* and *P. vivax*, children below age ten had low exposure compared to older individuals (> 70 years) [50]. In this study, the healthy Sabah controls (also from the northern part of Sabah) had higher *P. vivax* antibody levels compared to healthy controls from Bangkok, Rio De Janeiro and Melbourne, likely due to previous exposure to malaria, including *P. knowlesi*. A better understanding of the 21 *P. vivax* antigens, particularly the capability of antibodies against them to inhibit *P. knowlesi* invasion of RBCs in humans, is needed and should be explored further.

There are several limitations in this study. Firstly, the *P. vivax* antibodies used were previously chosen for their diagnostic utility as markers of recent past exposure to *P. vivax* infection; they were not specifically chosen for their immunological cross-protection against *P. knowlesi* red blood cell invasion, parasite multiplication or phagocytosis. Only three of the 21 antibodies had been previously tested and demonstrated to inhibit *P. knowlesi* invasion of red blood cells [22] and to protect from clinical *P. vivax* disease [17]. Nevertheless, these three *P. vivax* antibodies were associated with reduced *P. knowlesi* parasite counts. As such, although it is likely that we are predominantly measuring acute *P. knowlesi* responses, there is some degree of cross-protective *P. vivax* immunity present. We also only measured total IgG antibody responses and not markers of functional antibodies [51], which could be further explored in future studies. While the results from the machine learning methods underwent internal validation, it was not possible to perform an external data validation of the antibody measures selected in the univariate and machine learning analyses, limiting the generalisability of the findings. Finally, there are limitations in interpretation of the association between antibody levels and parasite clearance as it is unclear whether *P. vivax* antibodies are associated with parasite clearance time in *P. vivax* infections.

## Conclusions

Malaysia reports zero local cases of *P. falciparum* and *P. vivax*; however, the incidence of zoonotic *P. knowlesi* infections has increased and is now the leading cause of malaria. Declining exposure to nonzoonotic malaria may lead to reduced pre-existing immunity, potentially increasing the risk of clinical *P. knowlesi* cases. In this study, higher antibody responses to five *P. vivax* antigens were associated with lower parasitaemia; however, *P. vivax* cross-reactive antibody measures were not associated with *P. knowlesi* parasite clearance.

## Supplementary Material

Supplementary Files

This is a list of supplementary files associated with this preprint. Click to download.

- JThuraiRathnametalSupplementaryfigurestablesMalariaJ.docx

## Figures and Tables

**Figure 1:**
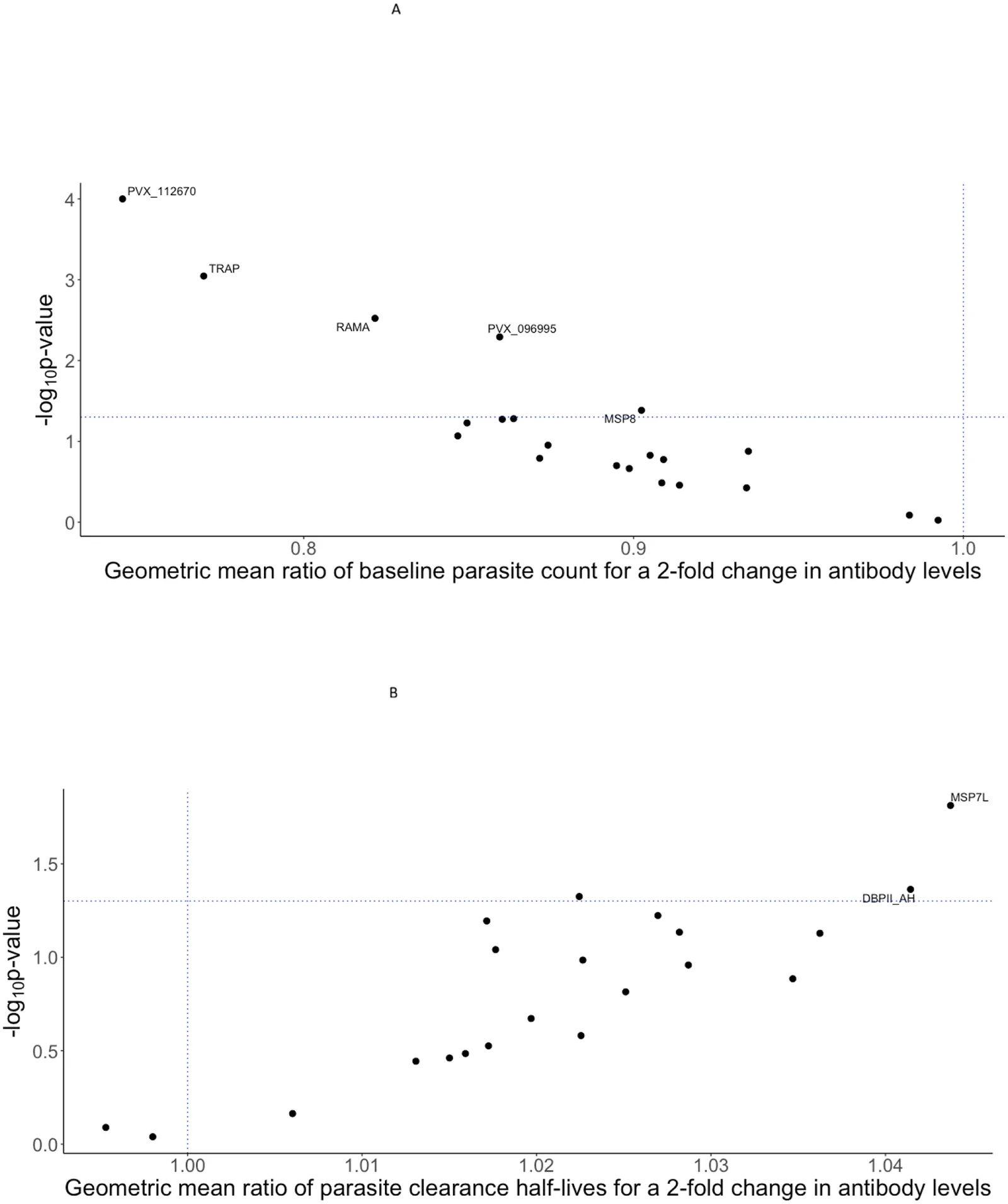
Volcano plots of the geometric mean ratio for **(A)** *P. knowlesi* parasite count at baseline, and **(B)** *P. knowlesi* parasite clearance half-lives, for a two-fold change in *P. vivax* antibody levels against the −log_10_ of the p-values from univariable linear regression models. The horizontal dotted line corresponds to a p-value of 0.05, while the vertical dotted line corresponds to a geometric mean ratio of one (i.e. no change in parasite count or clearance at baseline associated with a two-fold change in antibody level).

**Figure 2:**
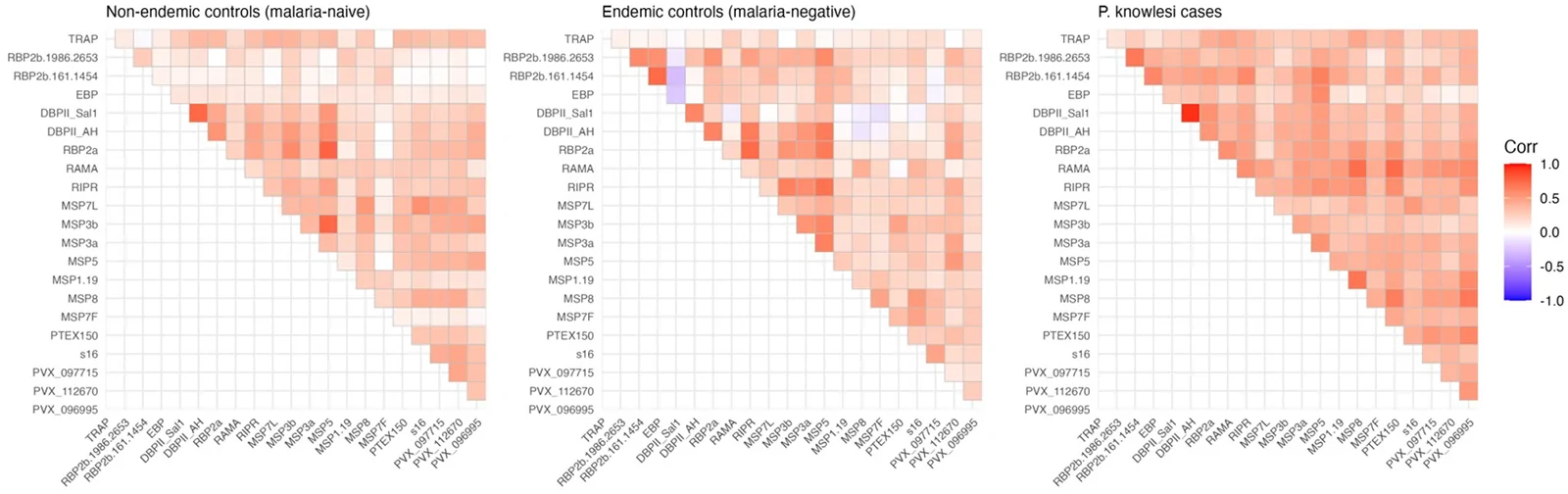
Correlation matrices of antibody responses. (A) Non-endemic malaria-naïve controls; (B) Endemic malaria-negative controls; (C) Acute *P. knowlesi* cases. Values are pairwise Spearman correlations.

**Table 1:** Clinical characteristics for participants.

Characteristics	*P. knowlesi* malaria cases N = 134	Healthy controls (non-*P. knowlesi* endemic area) N = 369^e^	Healthy controls (*P. knowlesi* endemic area) N = 112
**Gender**			
Female	34 (25%)	125 (34%)^e^	60 (54%)
Male	100 (75%)	77 (20%)^e^	52 (46%)
**Age (years)**			
Median, IQR^a^	37, 22–52	44, 33–57^e^	31, 26–43.5
Range (years)	3–78	18–77^e^	15–80
**Weight (kg** ^b^ **)**			
Median, IQR^a^	60.5, 49.9–71.3	-	-
Range (kg^b^)	11–103		
**Previous malaria**			
No	85 (63%)	-	-
Yes	49 (37%)		
**Days of preceding fever**			
Median, IQR^a^, Range (days)	5.5, 3–8, 0–14	-	-
**Parasitaemia (baseline)**			
Median, IQR^a^	2773, 662–8163	-	-
Range (/ μL of blood)	56–185,553		
**Parasite clearance half-life**			
Median, IQR^a^	2.6, 2.1–3.2	-	-
Range (hours)	1.0–9.9		
**Severe disease** ^c^			
No	130 (97%)	-	-
Yes	4 (3%)		
**Treatment**			
Artemisinin combination therapy (ACT)^d^	88 (66%)	-	-
Chloroquine	46 (34%)		

a IQR - inter-quartile range

b kg - kilograms

c WHO severe malaria research criteria [52]

d The treatment list includes artemether-lumefantrine, artesunate (intravenous) and artemether-lumefantrine (n = 8), and artesunate-mefloquine.

e No age and sex data available for n = 167 healthy controls from Thailand and Rio de Janeiro.

**Table 2:** Regression coefficient estimates (95% confidence interval) for the association of each *P. vivax* antibody feature (log_2_ transformed) with *P. knowlesi* parasite count at baseline (n = 134), adjusted for age and sex.

Antibody feature	Ratio of geometric mean of *P. knowlesi* parasite count at baseline (95% CI)	p-value
PVX_112670	0.7451 (0.6466, 0.8585)	< 0.01
TRAP	0.7697 (0.6612, 0.8959)	< 0.01
RAMA	0.8216 (0.7225, 0.9344)	< 0.01
PVX_096995	0.8594 (0.7737, 0.9548)	< 0.01
MSP8	0.9024 (0.8176, 0.9959)	0.04
RIPR	0.8636 (0.7448, 1.0016)	0.05
PTEX150	0.8602 (0.7383, 1.0022)	0.05
MSP7L	0.8495 (0.7171, 1.0064)	0.06
RBP2a	0.8467 (0.7001, 1.0240)	0.09
MSP3a	0.8741 (0.7403, 1.0320)	0.11
MSP1.19	0.9348 (0.8561, 1.0209)	0.13
RBP2b.1986.2653	0.9050 (0.7901, 1.0368)	0.15
DBPII_AH	0.8715 (0.7183, 1.0575)	0.16
MSP5	0.9091 (0.7936, 1.0414)	0.17
MSP7F	0.8948 (0.7546, 1.0612)	0.20
s16	0.8987 (0.7579, 1.0656)	0.22
DBPII_Sal1	0.9086 (0.7496, 1.1013)	0.33
EBP	0.9139 (0.7563, 1.1043)	0.35
RBP2b.161.1454	0.9343 (0.8030, 1.0870)	0.38
Hypothetical (PVX_097715)	0.9836 (0.8530, 1.1342)	0.82
MSP3b	0.9923 (0.7979, 1.2342)	0.94

**Table 3:** Model performance of each machine learning method used to analyse all 21 baseline *P. vivax* antibody measures simultaneously with and without inclusion of age, sex, and for parasite clearance outcome, initial *P. knowlesi* parasitaemia, and antimalarial drug treatment given (ACT or chloroquine).

Outcome	Machine learning method	Root mean squared error (RMSE) for all 21 antibodies without baseline demographic and clinical data	RMSE for all 21 antibody features with demographic and clinical data	Selected antibody features / Number of components^a^ (with demographic and clinical data factors)
*P. knowlesi* parasitaemia at baseline	Elastic net regression	1.617	1.618	PVX_096995 PVX_112670 TRAP
*P. knowlesi* parasitaemia at baseline	Random forest	1.648	1.655	PVX_112670 TRAP PVX_096995 DBPILAH MSP3b RAMA MSP7L MSP8
*P. knowlesi* parasite clearance	Elastic net regression	0.499	0.483	MSP7L DBPILAH s16 PVX_096995 MSP1.19
*P. knowlesi* parasite clearance	Random forest	0.510	0.498	MSP7L MSP3a MSP3b PTEX150 RBP2b.161.1454 RBP2b.1986.2653 RAMA RIPR

a -Ranked by variable importance derived from magnitude of regression coefficients for elastic net regression, and percentage increase in MSE for random forest method.

## Data Availability

The dataset analysed and other related materials used in this manuscript are available upon reasonable request.

## Abbreviations

ACT: artemisinin combination therapy
AL: artemether-lumefantrine
AS: artesunate
ASMQ: artesunatemefloquine
caret: classification and regression training
CI: Confidence interval
CQ: chloroquine
IgG: Immunoglobulin G
IQR: interquartile range
kg: kilogram
LASSO: least absolute shrinkage and selection operator
LOOCV: leave-one-out cross-validation
MSE: mean square error
NAI: naturally acquired immunity
*P. falciparum*: *Plasmodium falciparum*
*P. knowlesi*: *Plasmodium knowlesi*
*P. vivax*: *Plasmodium vivax*
PCR: polymerase chain reaction
RAU: relative antibody units
RBC: red blood cell
RCT: randomised controlled trial
RMSE: root mean square error
VIF: variance inflation factor
WHO: World Health Organisation
WWARN PCE: Worldwide Antimalarial Resistance Network Parasite Clearance Estimator
